# Perineurial Malignant Peripheral Nerve Sheath Tumor of the Cauda Equina: Diagnostic Challenge

**DOI:** 10.3390/diagnostics15212697

**Published:** 2025-10-24

**Authors:** Tomonori Kawasaki, Tomoaki Torigoe, Takuya Watanabe, Satoshi Kanno, Masataka Hirasaki, Arisa Kokubo, Kojiro Onohara, Masanori Wako, Tetsuhiro Hagino, Jiro Ichikawa

**Affiliations:** 1 Department of Pathology, Saitama Medical University International Medical Center, Hidaka 350-1298, Saitama, Japan; tomo.kawasaki.14@gmail.com (T.K.); kanno820@saitama-med.ac.jp (S.K.); ak1317@saitama-med.ac.jp (A.K.); 2 Department of Orthopaedic Oncology and Surgery, Saitama Medical University International Medical Center, Hidaka 350-1298, Saitama, Japan; ttorigoe@saitama-med.ac.jp (T.T.); tw52831@5931.saitama-med.ac.jp (T.W.); 3 Department of Clinical Cancer Genomics, Saitama Medical University International Medical Center, Hidaka 350-1298, Saitama, Japan; hirasaki@saitama-med.ac.jp; 4 Department of Radiology, Interdisciplinary Graduate School of Medicine, University of Yamanashi, Chuo 409-3898, Yamanashi, Japan; konohara@yamanashi.ac.jp; 5 Department of Orthopaedic Surgery, Interdisciplinary Graduate School of Medicine, University of Yamanashi, Chuo 409-3898, Yamanashi, Japan; wako@yamanashi.ac.jp (M.W.); tetsuhiro-hagino@outlook.jp (T.H.)

**Keywords:** malignant peripheral nerve sheath tumor, perineurial, cauda equina, imaging, histopathology

## Abstract

Malignant peripheral nerve sheath tumors (MPNSTs) are rare sarcomas with an extremely rare perineurial subtype. Herein, we present a case of a perineurial MPNST in the cauda equina. Clinically and radiologically, a mass extending from within the spinal canal at the L5 level to outside the intervertebral foramen was identified, raising suspicion of a neurogenic tumor as the primary diagnosis. Computed tomography-guided biopsy suggested an intermediate- to low-grade malignancy; however, a definitive diagnosis could not be established. Two years later, worsening neurological symptoms prompted further imaging, which revealed significant tumor growth and bone invasion. Open biopsy was performed to obtain a definitive diagnosis of perineurial MPNST. MPNSTs lack distinctive imaging features and are generally diagnosed based on a combination of radiological and histopathological findings. Although MPNSTs have a poor prognosis, the perineurial subtype is considered to have a relatively favorable outcome. Given these factors, early diagnosis followed by surgical resection or radiation therapy is recommended.

**Figure 1:**
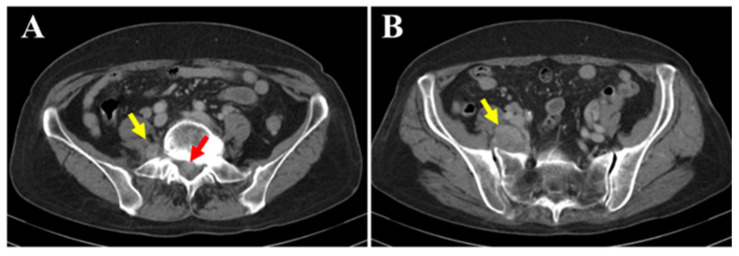
The patient was a 77-year-old woman who began experiencing pain from the right buttock to the lower limb three months prior to her first visit to our hospital. The patient initially consulted a local clinic, where imaging studies suggested a cauda equina tumor. A CT-guided biopsy was performed; however, the diagnosis remained challenging, and the patient was referred to our hospital. At her initial visit, she reported spontaneous pain and numbness extending from the right buttock to the posterior thigh and lower leg. There was no sensory dullness, the lower limb muscle strength was MMT grade 5, and no bladder or bowel dysfunction was observed. No café-au-lait spots were observed on the skin surface. Her medical history included hyperlipidemia and osteoporosis; however, she had no history of radiation therapy. Blood tests showed mildly decreased albumin (Alb 3.8 g/dL; normal range 4.1–5.1), slightly elevated creatinine (Cre 0.85 mg/dL; normal range 0.46–0.79), and mildly elevated IL-2R (597 U/mL; normal range 204–587), with all other values within normal limits. Contrast-enhanced CT (**A**,**B**) revealed a mass located both within the spinal canal (red arrow) and outside the intervertebral foramen (yellow arrow). No changes were observed in the surrounding bone.

**Figure 2:**
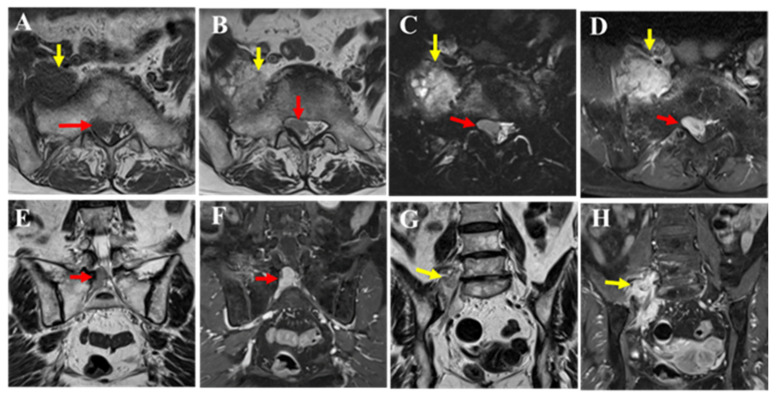
MRI revealed a mass extending from within the spinal canal at the L5/S1 level to the right intervertebral foramen (red arrow) and beyond the foramen (yellow arrow). The dural sac was displaced to the left of the spinal canal, indicating that the lesion was primarily extradural in nature. Compared to the muscle, the mass showed isointensity on T1-weighted images (**A**), mildly high signal intensity on T2-weighted images (**B**,**E**,**G**), and high signal intensity on T2 fat-suppressed images (**C**). Contrast enhancement was also observed (**D**,**F**,**H**). The portion of the mass outside the intervertebral foramen showed slightly heterogeneous internal characteristics on T2-weighted images (**B**,**G** yellow arrow), and nerve fibers were visible within it (**G**). Pathological findings from the CT-guided biopsy performed at the previous hospital suggested a spindle cell tumor. Although intermediate-to-low-grade malignancy was suspected, a definitive diagnosis could not be made. Therefore, an open biopsy was recommended to the patient; however, she declined, and the patient was followed conservatively. After two years, the patient experienced worsening pain and muscle weakness, leading to emergency hospitalization.

**Figure 3:**
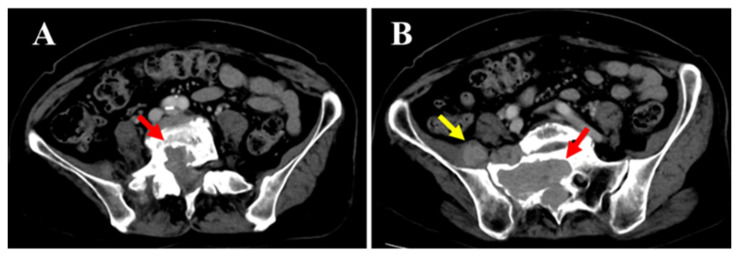
Spontaneous pain and numbness from the right buttock to the posterior thigh and lower leg worsened since the initial visit. Sensory dullness was mainly observed in the anterior lower leg and dorsum of the foot. The muscle strength in the lower limb was MMT grade 0 for both the tibialis anterior and extensor hallucis longus, while all other muscles remained at grade 5. Bladder or bowel dysfunction was not observed. Contrast-enhanced computed tomography (CT) (**A**,**B** the mass within the spinal canal (red arrow) and outside the intervertebral foramen (yellow arrow)) showed that the mass had increased in size over time and demonstrated bone invasion. Compressive changes resembling scalloping were also observed. No obvious metastasis to the other organs was observed.

**Figure 4:**
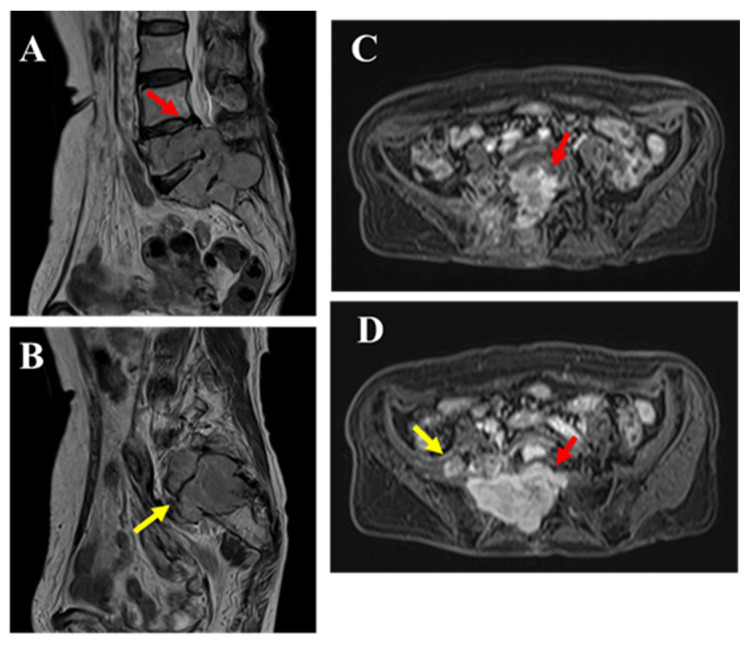
MRI revealed that although the mass had increased in size over time, its T2 signal intensity (**A**,**B** the mass within the spinal canal (red arrow) and outside the intervertebral foramen (yellow arrow)) and contrast enhancement (**C**,**D** the mass within the spinal canal (red arrow) and outside the intervertebral foramen (yellow arrow)) showed no significant changes compared to the initial imaging. Bone invasion was evident, and pathological fractures were observed in the affected bone. An open biopsy was performed to confirm the diagnosis.

**Figure 5:**
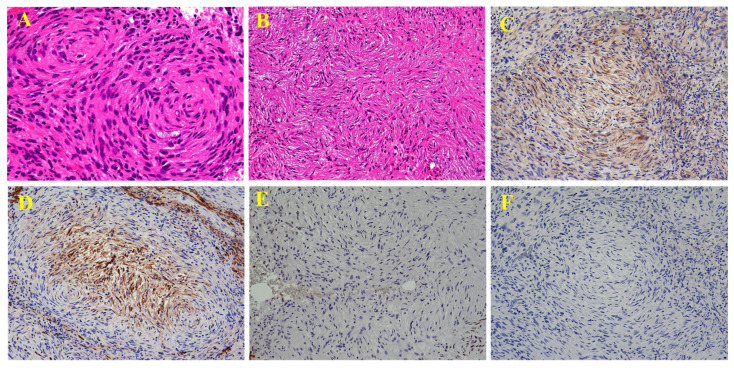
Histopathological examination revealed relatively uniform spindle-shaped tumor cells proliferating in fascicular, whorled ((**A** ×400), or storiform (**B**, ×200) patterns. The tumor cells exhibited characteristic bipolar elongated processes. Although the nuclear size and shape varied and irregularities were present, marked pleomorphism was not observed, and mitotic figures were few. Immunohistochemically, the tumor cells were positive for EMA (**C**, ×200) and Glut-1 (**D**, ×200), showed loss of H3K27me3 expression (**E**, ×200), and were negative for S-100 (**F**, ×200). The Ki-67 (MIB-1) labeling index was 20% in hot spots. Based on these findings, the diagnosis was perineurial malignant peripheral nerve sheath tumor (MPNST), classified as grade 1 according to the FNCLCC Grading System. The patient declined surgery and decided to undergo carbon-ion radiotherapy. MPNST is a malignant spindle cell tumor originating from peripheral nerves and accounts for approximately 3–5% of all malignant soft-tissue tumors. It typically occurs in individuals aged 20–50 years old. In patients with neurofibromatosis type 1 (NF1), onset tends to occur earlier than in sporadic cases [1,2]. Common sites of occurrence include the limbs and trunk; however, MPNST arising from the cauda equina is extremely rare, with only 15 reported cases [3]. There is little to no sex difference in the incidence, although some reports have suggested a slight male predominance [2,4]. Symptoms vary widely, from pain and numbness, as seen in this case, to almost painless presentations, depending on the relationship between the tumor and nerve [1]. Reports of cases complicated by subarachnoid hemorrhage [5] and metastatic MPNST to the cauda equina also exist [6], indicating that a full-body examination may be necessary. Background factors such as the presence of NF1 and a history of radiation therapy are important [1]. The reported rate of NF1 comorbidities in patients with MPNST varies, with some studies reporting rates as high as 66% [4]. Additionally, 8–13% of patients with NF1 develop MPNST [5]. The frequency of radiation-induced MPNST varies, with some reports suggesting a rate of up to 10% [2]. Similar to the usual MPNST, sporadic [7], NF1-associated [5], and radiation-induced [3] cases of MPNST of the cauda equina have been reported; however, no comprehensive studies have been conducted, and the details remain unclear. MRI findings of MPNST may include heterogeneous signals on T1- and T2-weighted images, contrast enhancement, intratumoral cystic changes, surrounding edema, and well-defined margins. However, these features vary in frequency and are not specific to MPNST, as they are also observed in other sarcomas [8,9]. The differentiation between neurofibromas and MPNST is challenging. On MRI, differences in size, shape, signal intensity, split fat sign, target sign, edema, and enhancement patterns, as well as SUV_max_ values on PET, have been reported to be useful for differentiation [10,11]. In addition, the coefficient of variation in T2-weighted images has been reported to correlate with overall survival. In the present case, a neurogenic tumor was suspected. However, accurately distinguishing between benign and malignant tumors based on imaging alone is impossible, particularly in sporadic cases. From the perspective of intraspinal tumors, differential diagnoses beyond nerve-related tumors, such as MPNSTs, including astrocytomas, ependymomas, lymphomas, and metastatic tumors, should be considered [12]. Considering the challenges associated with imaging-based diagnosis, histopathological examination is critical. MPNST is pathologically difficult to diagnose because of the lack of distinctive morphological and immunohistochemical findings, making the patient’s history of NF1 and the tumor’s neurogenic origin important diagnostic clues [2]. Grossly, tumors often arise within or around the nerves. Microscopically, they exhibit varying degrees of cellular morphology and possess high invasive potential [2]. MPNST has several subtypes, including epithelioid, triton, and perineurial variants [1,13]. Perineurial MPNSTs show perineurial cell differentiation and are characterized by EMA and GLUT-1 positivity and S100 negativity on immunohistochemistry. They are reported to have a better prognosis than other subtypes and are not associated with NF1 [2]. Perineurial MPNSTs have a wide age range of onset (11–83 years) and commonly arise in the limbs, although cases originating in the stomach have also been reported [13,14]. Many cases lack continuity with the nerves [13]. Pathologically, MPNSTs must be carefully distinguished from a wide range of other tumors, including synovial sarcoma, angiosarcoma, carcinoma, melanoma, and myoepithelial tumors; however, there are no IHC markers that specifically identify MPNST among these tumors [2]. For example, S100 shows focal positivity in approximately 50% of cases. Epithelial markers, such as AE1/AE3 and EMA, were also focally positive. CD34 is positive in approximately 25% and 30% of cases, respectively [2]. The loss of H3K27me3 has recently been reported as a useful marker for diagnosing MPNST. However, because the positivity rate is approximately 70%, and similar findings can be seen in melanocytic tumors, caution is required when using this marker for diagnosis [15]. Surgical resection is the primary treatment approach, with R0 resection (complete removal of the tumor, both macroscopically and microscopically) being the recommended goal. In cases such as this one, achieving R0 resection may be difficult, and there is a risk of bladder and rectal dysfunction; combined or even standalone radiotherapy may be considered. Although it is useful for local control, its effect on overall survival remains unclear [11]. Carbon ion radiotherapy has been increasingly used to treat various sarcomas, and its effectiveness in MPNST has also been reported [16]. In the present case, carbon ion radiotherapy was administered. However, because no comparative trials with conventional radiation therapy exist, further analysis is required to determine the most effective treatment. Chemotherapy with agents such as ifosfamide and anthracyclines has been reported; however, their effects on survival and local control are limited. For low-risk patients, chemotherapy is generally not recommended, and the development of new therapeutic agents is eagerly awaited [11]. The recurrence rate is estimated to be 30–40%, with 5-year survival rates ranging from 26 to 60% and 10-year survival rates of approximately 45% [2,4]. The prognostic factors included tumor size, presence of NF1, location, and grade [1]. In this report, we present the first case of perineurial MPNST arising from the cauda equina and discuss its diagnosis and treatment.

## Data Availability

The data presented in this study are available from the corresponding author upon request. The data are not publicly available due to privacy restrictions.

## Abbreviations

The following abbreviations are used in this manuscript:

MRI	Magnetic resonance imaging
MPNST	Malignant peripheral nerve sheath tumor
CT	Computed tomography
NF1	Neurofibromatosis type 1
